# Prognostic value of prognostic nutritional index in patients with hepatocellular carcinoma undergoing hepatectomy: a systematic review and meta-analysis

**DOI:** 10.3389/fonc.2026.1887443

**Published:** 2026-07-29

**Authors:** Zimo Gao, Zhichen Kang, Shengpeng Yao, Xinghai Chen

**Affiliations:** 1 Department of Emergency Medicine, The Second Hospital of Jilin University, Changchun, China; 2 Department of Rehabilitation, The Second Hospital of Jilin University, Changchun, China; 3 Department of Thyroid Surgery, The Second Hospital of Jilin University, Changchun, China; 4 Department of Intensive Medicine, The Second Hospital of Jilin University, Changchun, China

**Keywords:** hepatectomy, hepatocellular carcinoma, meta-analysis, PNI, prognostic nutritional index

## Abstract

**Objective:**

To evaluate the value of preoperative prognostic nutritional index (PNI) in predicting the long-term survival of patients with hepatocellular carcinoma (HCC) after hepatectomy through systematic review and meta-analysis.

**Methods:**

PubMed, Embase, Web of Science, and Cochrane Library were searched to collect observational studies on the association between preoperative PNI and prognosis of HCC patients after hepatectomy from inception to April 2026. Two researchers independently screened the literature and extracted the data, and the Newcastle-Ottawa Scale (NOS) was used to assess the risk of bias of the included studies. A random-effects model was used to evaluate the effect of PNI on overall survival (OS), disease-free survival (DFS) and recurrence-free survival (RFS). Sensitivity and subgroup analyses were performed to test the stability of the results, and Egger’s test was used to assess publication bias.

**Results:**

A total of 16 studies were included. The results of the meta-analysis showed that higher preoperative PNI was associated with significantly improved OS (pooled HR = 0.44, 95%CI: 0.29-0.65, P < 0.001) and DFS (pooled HR = 0.67, 95%CI: 0.50-0.90, P = 0.007) and RFS (pooled HR = 0.76, 95%CI: 0.69-0.84, P < 0.001). Sensitivity analysis showed that the pooled results did not change direction after removing individual studies one by one. Egger’s test suggested that publication bias may exist in the OS analysis, but the association between PNI and OS remained statistically significant after the correction (adjusted HR = 0.35, 95%CI: 0.17-0.75). Subgroup analysis results suggest that the PNI cutoff may be the one of the source of heterogeneity.

**Conclusion:**

Current evidence suggests that a higher preoperative prognostic nutritional index is a potential protective factor for longer overall survival, disease-free survival and recurrence-free survival in patients with HCC after curative hepatectomy. As an easily accessible composite index, PNI may serve as a complementary tool for preoperative risk assessment, but its incremental predictive value over established prognostic models requires further validation in prospective studies.

**Systematic review registration:**

https://www.crd.york.ac.uk/PROSPERO/view/CRD420261389510, identifier CRD420261389510.

## 1 Introduction

Hepatocellular carcinoma (HCC) is one of the most common malignant tumors worldwide, and its high incidence and mortality rates pose a serious threat to human health (1, 2). Radical hepatectomy remains the preferred treatment for early-stage and some intermediate-stage HCC patients, offering a potential cure (3). However, HCC patients still face a high risk of recurrence and metastasis after surgery, significantly impacting their long-term survival (4). Therefore, accurately identifying the key factors influencing the prognosis after HCC hepatectomy is of crucial clinical significance for improving overall patient survival. Among the many potential influencing factors, the patient’s systemic status, particularly nutritional and immune status, is receiving increasing attention (5, 6). Tumor growth and progression are not isolated events but involve intricate interactions with systemic inflammatory responses and nutritional status (7, 8). These interactions directly or indirectly affect the biological behavior of the tumor, its response to treatment, and the patient’s ultimate prognosis (9).

Against this backdrop, biomarkers that comprehensively reflect the host’s nutritional and immune status have become a research hotspot (10, 11). The Prognostic Nutrition Index (PNI) is a simple, readily available, and cost-effective composite indicator (12). This index assesses both the body’s nutritional reserves and cellular immune status by integrating serum albumin concentration and peripheral blood lymphocyte count (13, 14). Low albumin levels suggest malnutrition and impaired synthetic function (15), while low lymphocyte counts may reflect a weakened anti-tumor immune response (16). Therefore, PNI is considered an important bridge connecting a patient’s overall condition with tumor prognosis (17). In recent years, the prognostic value of PNI in various solid tumors, such as gastric cancer (18), colorectal cancer (19), and pancreatic cancer (20), has been widely validated. Although a considerable number of observational studies have explored the association between preoperative PNI and postoperative survival in HCC patients, these studies show inconsistencies (12, 17, 21). This inconsistency may stem from differences in sample size, population characteristics, PNI cutoff values used, and statistical models among the studies. This makes it difficult for clinicians to reach a consensus on the clinical application value of PNI and hinders its integration into clinical decision-making processes.

While previous meta-analyses have explored the prognostic value of PNI in patients undergoing hepatectomy for hepatocellular carcinoma (22), our meta-analysis represents a significant advancement and innovation in methodological rigor, the timeliness of evidence, and the depth of analysis. Firstly, we updated the search period to April 2026 to capture the latest developments in the field. Secondly, we added six new, more methodologically sound studies published between 2024 and 2026 (12–14, 17, 21, 23), after excluding low-quality studies. This allows our analysis to be based on a higher-quality and more novel set of evidence. We hypothesize that by including these recent, high-quality studies and applying more rigorous analytical methods, we can provide a more robust and reliable summary of contemporary evidence regarding the prognostic value of PNI, thereby addressing potential shortcomings in the quality and timeliness of evidence in previous studies.

## 2 Methods

### 2.1 Literature search

This meta-analysis was conducted in accordance with the PRISMA 2020 statement (24) and was registered in PROSPERO (registration number: CRD420261389510). The PRISMA 2020 checklist is provided in the **Supplementary Materials**. To identify relevant studies assessing the prognostic value of PNI in patients with HCC undergoing hepatectomy, a systematic literature search was performed in PubMed, Embase, Web of Science, and the Cochrane Library up to April 2026. The search query in PubMed is as follows: ((“Carcinoma, Hepatocellular”[Mesh]) OR ((((Hepatocellular Carcinoma) OR (Hepatoma)) OR (Adult Liver Cancer)) OR (Liver Cell Carcinoma))) AND ((prognostic nutritional index[Title/Abstract]) OR (PNI)). Detailed search strategies of all databases are presented in **Supplementary Table 1**. To ensure completeness, the bibliographies of all included articles were manually screened. The process of article selection and evaluation was conducted independently by two investigators, and any discrepancies were resolved through discussion.

### 2.2 Inclusion and exclusion criteria

Inclusion criteria:

P: Patients diagnosed with HCC and who underwent hepatectomy.
E: High PNI.
C: Low PNI.
O: Any survival outcome, including overall survival (OS), disease-free survival (DFS) and recurrence-free survival (RFS), etc. The OS is defined as the time from surgery to any cause of death; the DFS is defined as the time from surgery to recurrence, metastasis or any cause of death; the RFS is defined as the time from surgery to the imaging confirmation of tumor recurrence.
S: Study design was cohort or case-control.

Exclusion criteria: Studies that did not meet the following criteria were excluded: protocols, unpublished data, and non-original publications including letters, abstracts, commentaries, corrections, and replies. Single-arm trials, review articles, and studies with insufficient data-particularly those in which hazard ratios (HRs) and 95% confidence intervals (CIs) for survival outcomes could not be extracted or calculated-were also omitted.

### 2.3 Data abstraction

Data extraction was performed independently by two investigators, with any disagreements adjudicated by a senior researcher. The extracted data encompassed the first author’s name, publication year, geographic region, study design, population characteristics, sample size, patient demographics, cutoff values, as well as HRs and 95% CIs for OS, RFS, and DFS. Corresponding authors were contacted to obtain any missing or unclear data. The PNI was calculated as: serum albumin (g/L) + 5 × peripheral blood lymphocyte count (×10^9^/L).

### 2.4 Quality evaluation

The quality of the included observational studies (cohort and case-control designs) was appraised with the Newcastle-Ottawa Scale (NOS) (2). A predefined threshold score of 7 to 9 was used to classify studies as high quality (25). This appraisal followed a dual-reviewer process conducted independently, with any divergences in scoring resolved by consensus discussion.

### 2.5 Statistical analysis

The analysis was performed with Review Manager software (v5.4.1). Survival data were synthesized using pooled HRs with 95% CIs, based on a random-effects model. Study heterogeneity was quantified with Cochrane’s Q test and the I² inconsistency index (26, 27). A threshold of P < 0.10 for the Q test and I² > 50% defined high heterogeneity. Sensitivity analysis was employed for outcomes derived from more than two studies to evaluate the robustness of the results. Subgroup analyses, stratified by cutoff value, were conducted to investigate sources of significant heterogeneity. Potential publication bias was examined using funnel plots and Egger’s regression test (outcomes included ≥10 studies) (28) in Stata 15.1; a P value < 0.05 was considered statistically significant. For outcomes showing evidence of publication bias, the trim-and-fill method was used to adjust the estimates. Additionally, according to GRADE, each outcome’s evidence was evaluated and graded as “high”, “moderate”, “low”, or “very low” quality (29).

## 3 Results

### 3.1 Literature retrieval and study characteristics

**Figure 1:**
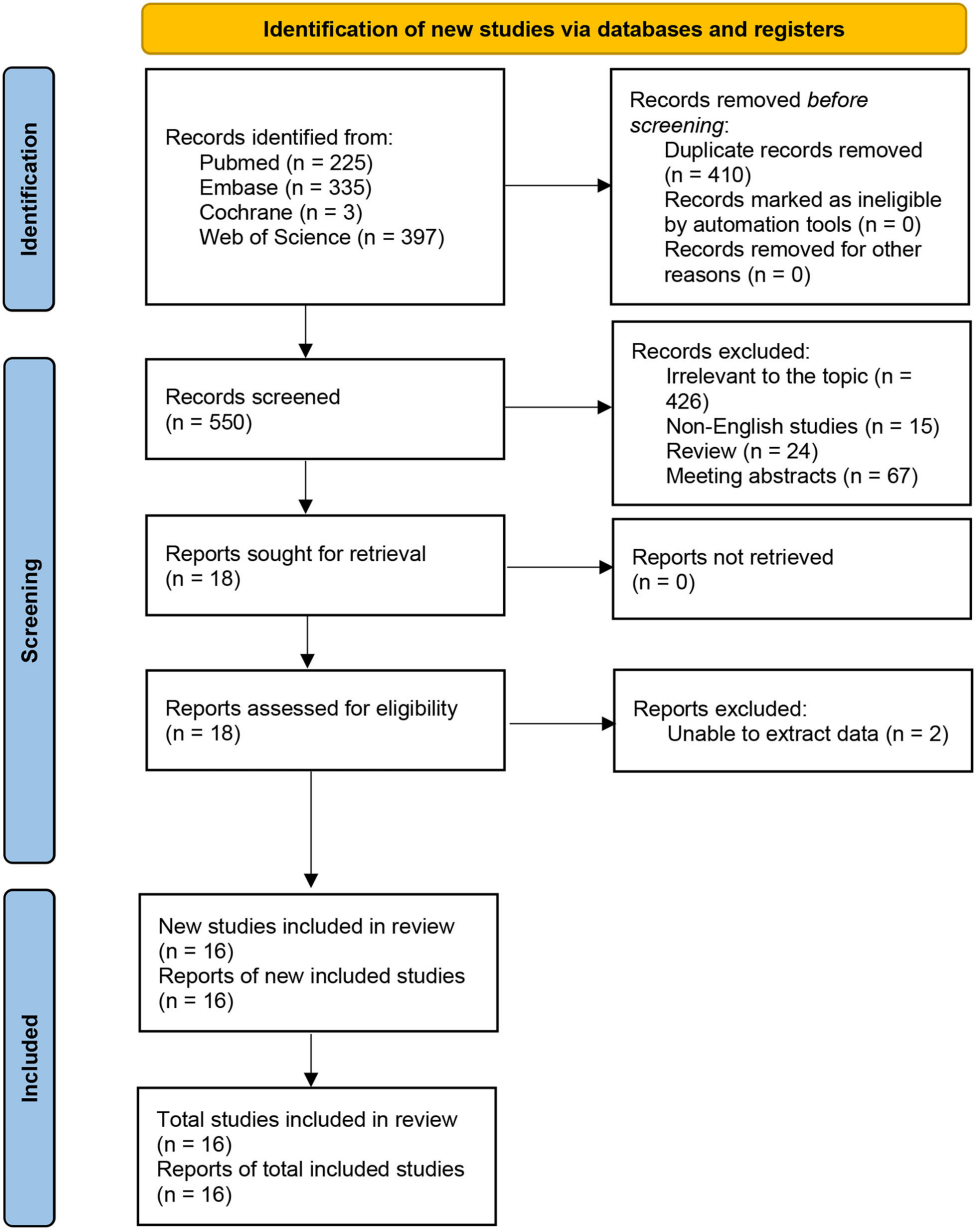
Flowchart of the systematic search and selection process.

The process of literature identification and selection is illustrated in **Figure 1**. A comprehensive search across PubMed (n = 225), Embase (n = 335), Web of Science (n = 397), and the Cochrane Library (n = 3) initially yielded a total of 960 records. After duplicate entries were removed, 550 unique titles and abstracts were screened for relevance. In the final analysis, 16 studies encompassing a total of 4685 patients were deemed eligible and included in the meta-analysis (12–14, 17, 21, 23, 30–39). **Table 1** summarizes the key features and methodological quality of each eligible study. The included literature was published between 2021 and 2026.

**Table 1:** Characteristics and quality assessment of included studies.

Author	Region	Study design	Population	No. of patients	Gender		Mean/median age	Child-Pugh score A/B/C	HBV	Cirrhosis	PNI cut-off	Median follow-up	NOS
					Male	Female							
Cheng (17)	China	Retrospective	Patients with HCC who underwent curative resection	187	156	31	60	NA	173	145	44	1082 days	7
Fan (30)	China	Retrospective	HCC patients with hepatectomy	279	NA	NA	51	NA	NA	258	51.9	NA	7
Hayashi (31)	Japan	Retrospective	Patients who underwent curative hepatectomy for HCC	303	221	82	70.5	295/8/0	49	NA	46.2	NA	7
Jeong (32)	Korea	Retrospective	Patients who underwent curative hepatectomy for hepatocellular carcinoma	130	111	19	57.5	118/12/0	85	NA	52	34.5 months	8
Liang (33)	China	Retrospective	Patients diagnosed with HBV-HCC and undergoing radical hepatectomy	868	727	141	NA	NA	NA	NA	46	NA	7
Mao (34)	China	Retrospective	Hepatocellular carcinoma after surgical resection	360	298	62	59.2	354/6/0	311	173	NA	24 months	7
Matsumoto (35)	Japan	Retrospective	Patients diagnosed HCC using the Milan criteria who underwent hepatic resection	497	374	123	69	460/37/0	106	NA	45	51.2 months	7
Qian (36)	China	Retrospective	HCC patients with hepatectomy	661	572	89	51	621/40/0	555	477	45	36 months	7
Qu (37)	China	Retrospective	Patients who underwent curative resection for HCC	215	178	37	59.1	188/27/0	160	NA	43.75	31 months	8
Su (14)	China	Retrospective	Patients with HCC undergoing hepatectomy	285	244	41	57	205/79/0	243	198	44.4	15 months	8
Tanemura (23)	Japan	Retrospective	Patients with primary HCC who underwent initial hepatectomy	151	126	25	69	NA	19	38	NA	34 months	7
Tsukagoshi (21)	Japan	Retrospective	Patients with HCC who underwent initial hepatic resection	203	166	37	72	197/NA/NA	22	NA	45	39.2 months	7
Wu (38)	China	Retrospective	Hepatocellular Carcinoma After Partial Hepatectomy	88	62	26	NA	NA	68	37	44.35	NA	7
Wu (13)	China	Retrospective	HCC patients after radical resection	120	104	16	70.4	NA	NA	77	53.1	NA	9
Yugawa (39)	Japan	Retrospective	Patients who underwent hepatic resection for HCC	120	84	36	70	119/1/0	17	58	46.5	NA	7
Zhang (12)	China	Retrospective	Patients with HCC receiving curative-intent hepatectomy	218	178	40	55	NA	NA	135	46.18	NA	7

### 3.2 Prognostic value of PNI for OS

**Figure 2:**
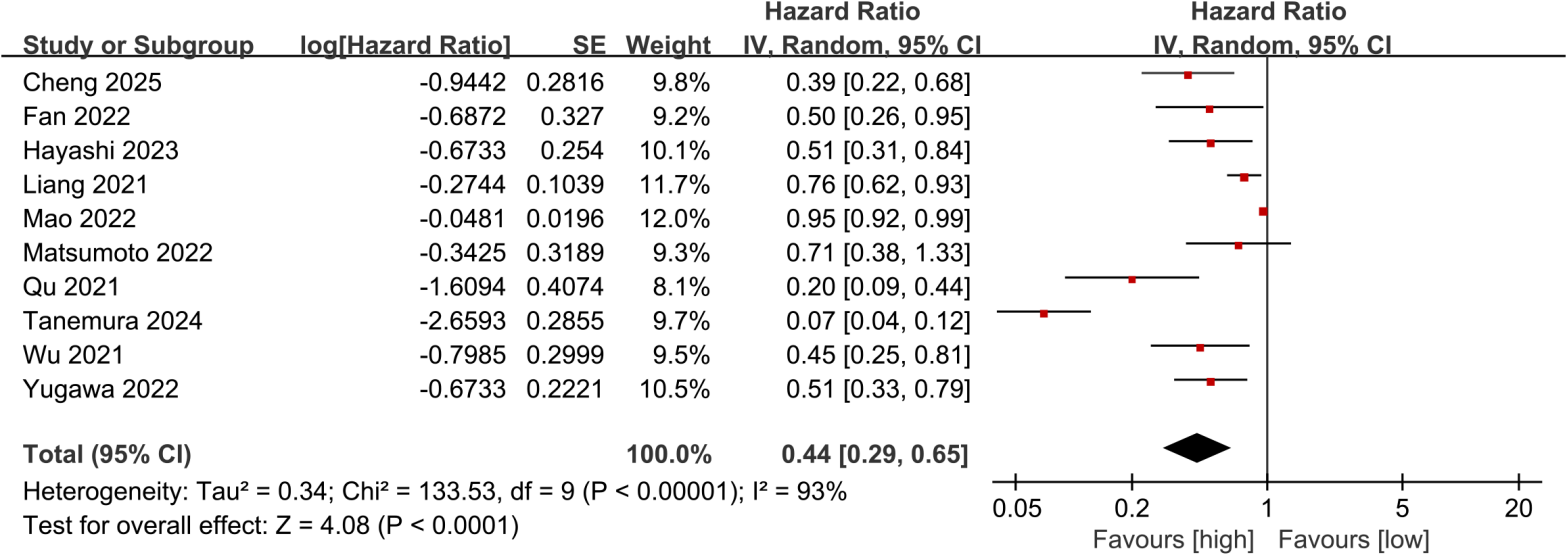
Forest plots of the prognostic value of PNI for OS.

**Figure 3:**
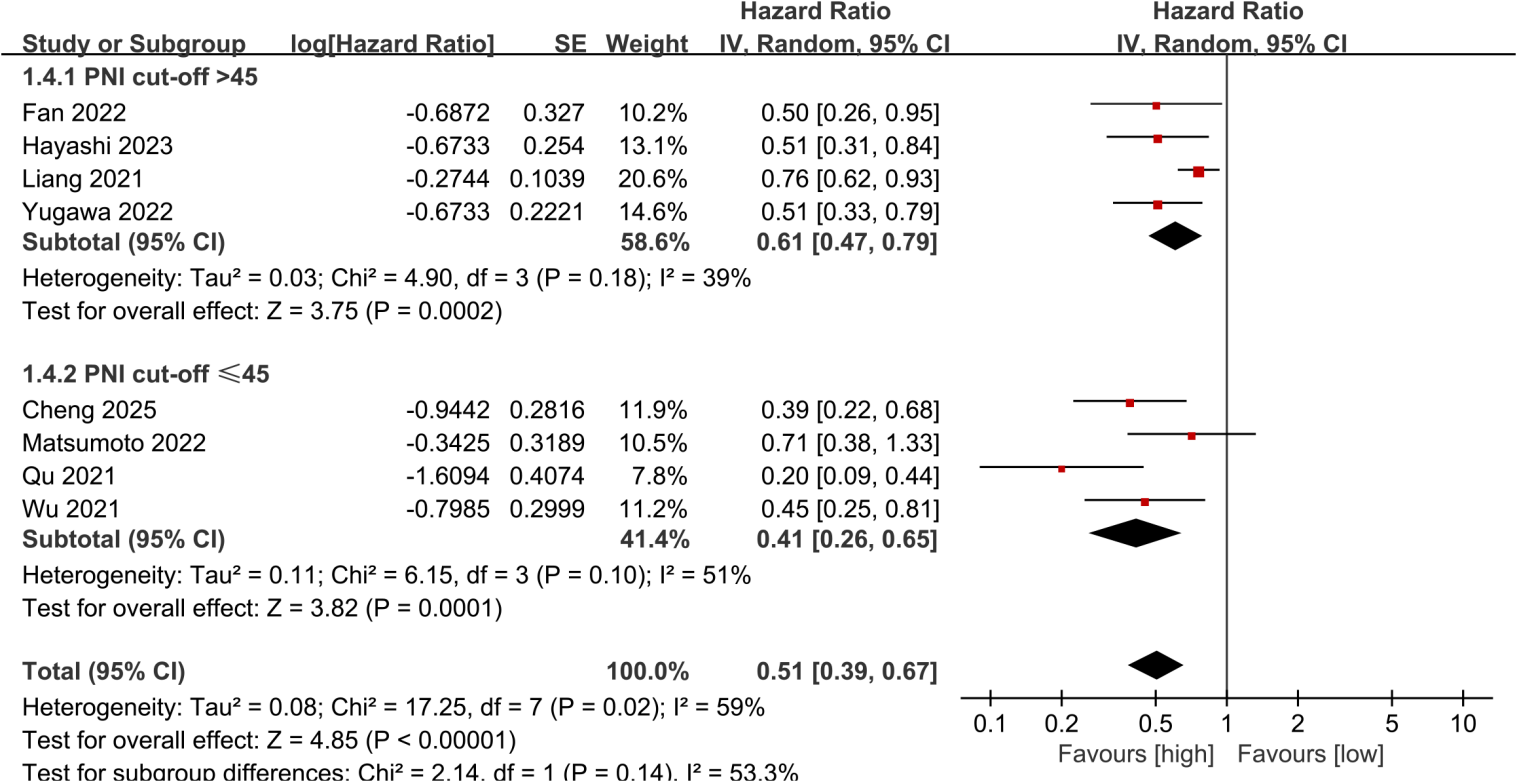
PNI cutoff-based subgroup analysis of the prognostic value of PNI for OS.

OS results were synthesized from 10 studies. The meta-analysis indicated that a higher PNI was significantly associated with longer OS (HR: 0.44; 95% CI: 0.29, 0.65; *P* <0.0001), with a substantial heterogeneity (*I*^2^ = 93%, *P* <0.00001) (**Figure 2**). Subgroup analysis of OS based on the PNI cutoff value revealed that the correlation between PNI and OS remained significant in both the cutoff value > 45 and the cutoff value ≤ 45 subgroups (**Figure 3**). Heterogeneity decreased to 39% and 51% in the subgroups with PNI cutoff < 45 and ≥ 45, respectively (**Figure 3**).

### 3.3 Prognostic value of PNI for RFS

**Figure 4:**
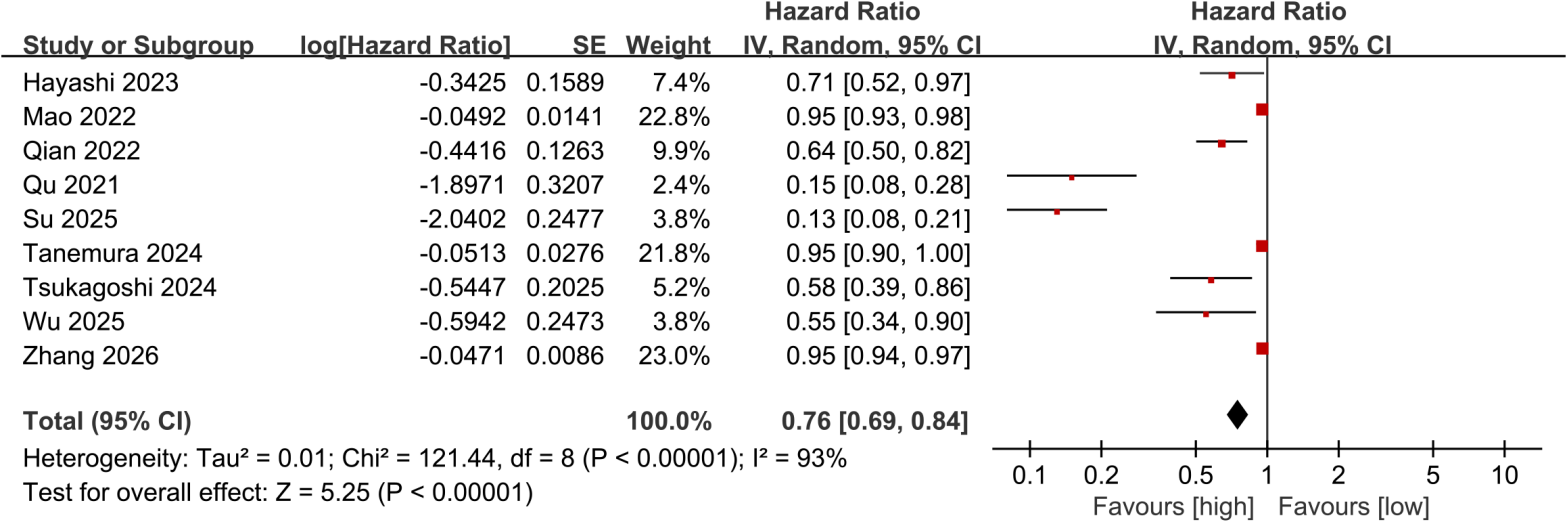
Forest plots of the prognostic value of PNI for RFS.

RFS results were synthesized from 9 studies. The meta-analysis indicated that a higher PNI was significantly associated with longer RFS (HR: 0.76; 95% CI: 0.69, 0.84; *P* <0.00001), with a substantial heterogeneity (*I*^2^ = 93%, *P* <0.00001) (**Figure 4**).

### 3.4 Prognostic value of PNI for DFS

**Figure 5:**
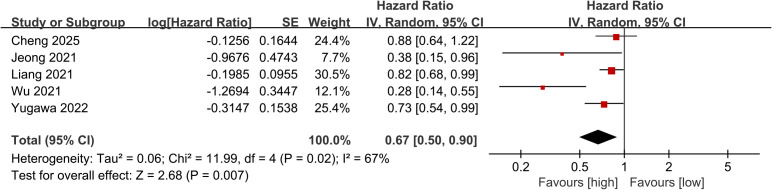
Forest plots of the prognostic value of PNI for DFS.

DFS results were synthesized from 5 studies. The meta-analysis indicated that a higher PNI was significantly associated with longer DFS (HR: 0.67; 95% CI: 0.50, 0.90; *P* = 0.007), with a substantial heterogeneity (*I*^2^ = 67%, *P* = 0.02) (**Figure 5**).

### 3.5 Publication bias

**Figure 6:**
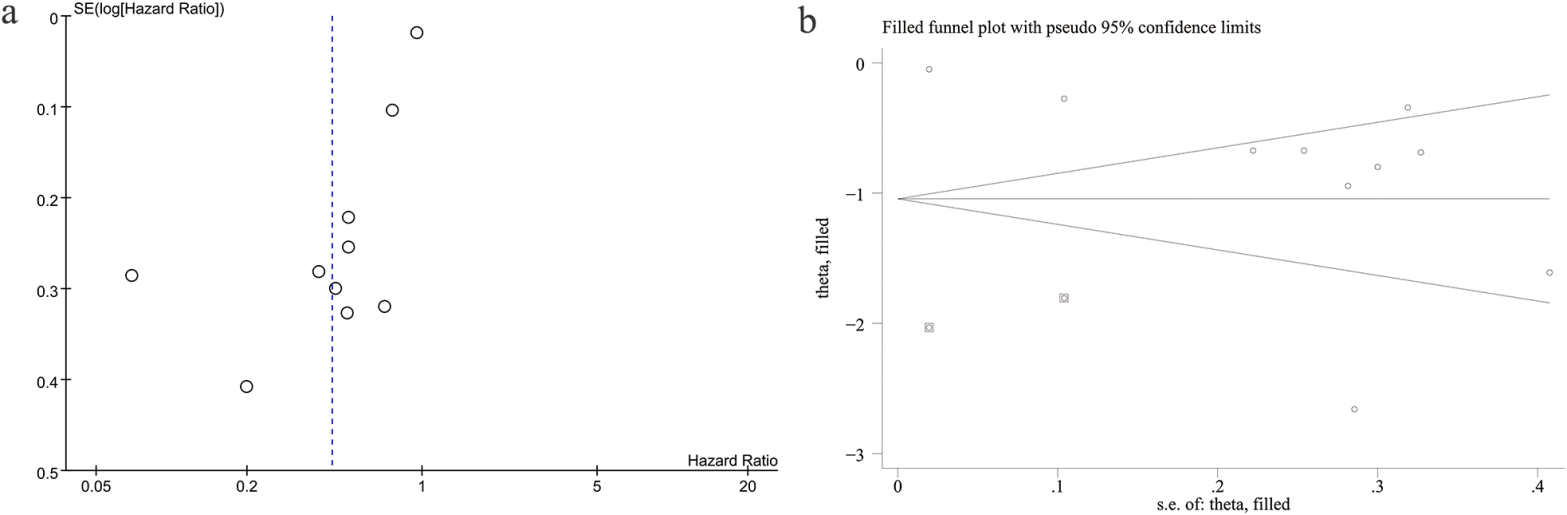
**(A)** Funnel plot of OS, **(B)** trimming and filling funnel plot of OS.

The potential for publication bias concerning the prognostic value of PNI for OS was examined using funnel plot analysis and Egger’s regression test. These analyses revealed significant publication bias for OS (Egger’s test P = 0.004, **Figure 6A**). The influence of this bias on the pooled outcome was further evaluated with the trim-and-fill method. After performing the trim-and-fill adjustment, the significant association between PNI and OS remained unchanged, indicating that the overall result for OS was not substantially affected by publication bias (adjusted HR: 0.35; 95% CI: 0.17–0.75) (**Figure 6B**).

### 3.6 Sensitivity analysis

**Figure 7:**
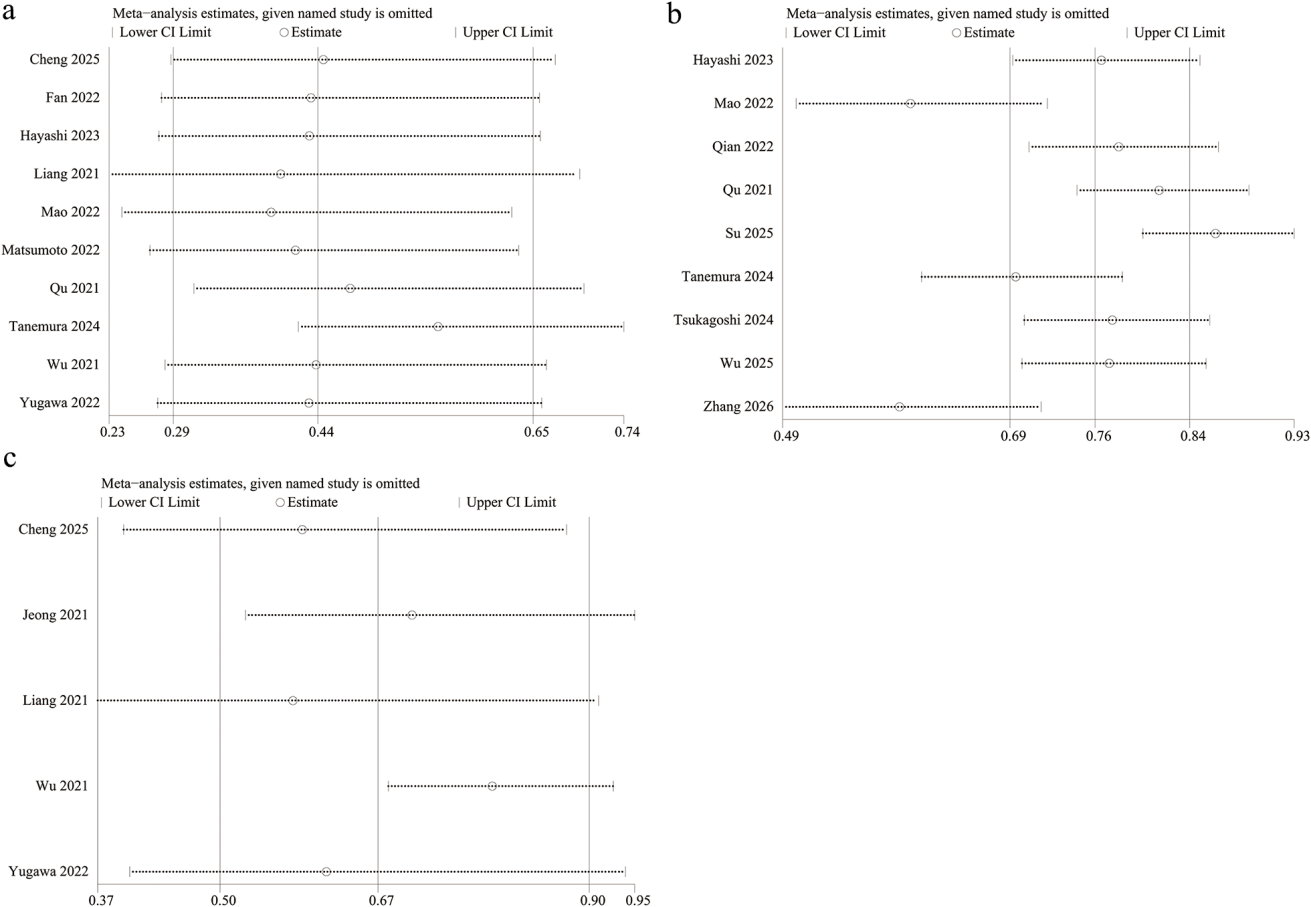
Sensitivity analysis of the prognostic value of PNI for **(A)** OS, **(B)** RFS, and **(C)** DFS.

To evaluate the robustness of the pooled results, a sensitivity analysis using the leave-one-out method was conducted. This analysis sequentially excluded each study to assess its individual influence on the overall HRs for OS, RFS, and DFS. The summary HRs for OS (**Figure 7A**), RFS (**Figure 7B**), and DFS (**Figure 7C**) were not substantially altered by the omission of any single study, indicating the stability of the findings.

### 3.7 GRADE rating

The GRADE system was used to assess the certainty of evidence for the primary outcome measures in this meta-analysis. The initial rating was set as ‘low’ quality because all included studies were retrospective observational studies. For OS, due to extremely high heterogeneity and significant publication bias indicated by Egger’s test, the quality of evidence was downgraded twice, ultimately being rated ‘very low’ quality. For RFS, it was also downgraded once due to extremely high heterogeneity, ultimately being rated ‘very low’ quality. For DFS, it was downgraded once due to moderate heterogeneity, ultimately being rated ‘very low’ quality. The specific reasons for the downgrades are detailed in the GRADE evidence summary table in **Table 2**.

**Table 2:** GRADE rating of each outcome.

No. of studies	Outcomes	HR	95%CI	I2; P value	Risk of bias	Inconsistency	Indirectness	Imprecision	Publication bias	Plausible confounding	Magnitude of effect	Dose-response gradient	GRADE
10	OS	0.44	0.29, 0.65	93%; P <0.00001	No serious risk	Serious inconsistency	No serious indirectness	No seriousimprecision	Strongly suspected	Would notreduce effect	Yes	No	Very low
9	RFS	0.76	0.69, 0.84	93%; P <0.00001	No serious risk	Serious inconsistency	No serious indirectness	No seriousimprecision	NA	Would notreduce effect	No	No	Very low
5	DFS	0.67	0.50, 0.90	67%; P=0.02	No serious risk	Serious inconsistency	No serious indirectness	Seriousimprecision	NA	Would notreduce effect	No	No	Very low

## 4 Discussion

This study synthesized data from 16 observational studies involving 4685 patients through systematic retrieval and meta-analysis. Our main finding suggests that a higher preoperative PNI is a potential protective factor for longer OS, DFS, and RFS. This result provides potential evidence supporting the application of PNI as a valuable prognostic biomarker in HCC surgical clinical practice. The findings of our study are consistent with the conclusions of the previous meta-analysis (22). However, previous studies included relatively early literature, possibly including abstracts from non-English journals or conferences. The methodological details and reporting standards of these studies may vary. The six recent studies added to this study represent the latest research in the field. New studies generally follow more modern reporting standards and may have been more adequately adjusted for potential confounding factors. Including these high-quality new studies enhances the timeliness and methodological strength of the evidence. At the analytical level, previous studies have indicated that the PNI cutoff value is an important source of heterogeneity (22). Similarly, our subgroup analysis further focused on and confirmed that the PNI cutoff value is a core and actionable factor contributing to heterogeneity. This finding provides a clearer basis for unifying or standardizing the application threshold of PNI in future studies. Therefore, this study can be seen as an important update and quality improvement to previous meta-analyses, providing evidence with greater timeliness and methodological assurance for clinical decision-making.

Although PNI showed clear overall prognostic value, our analysis also observed significant heterogeneity. This heterogeneity suggests that the strength of the association between PNI and prognosis may be moderated by other factors. Our subgroup analysis focused on the cutoff value of PNI to explore the sources of heterogeneity. The analysis found that intragroup heterogeneity was significantly reduced when subgroup analyses were performed according to different cutoff values (>45 and ≤45). Meanwhile, the significant association between PNI and OS remained unchanged in both subgroups. This result suggests that the difference in PNI cutoff values ​​used in the studies is the cause of overall heterogeneity. This difference may stem from differences in demographic characteristics, tumor biological behavior, baseline liver function, and statistical methods among different study populations (12, 14, 21, 31, 35). Future studies with larger sample sizes or standardized methods are needed to explore and validate the optimal PNI cutoff value for HCC hepatectomy patients to enhance the standardization and comparability of its clinical application.

In assessing publication bias, we noted that the Egger test for OS suggested potential publication bias, a common limitation in meta-analyses of observational studies. To evaluate the potential impact of this bias on the robustness of our conclusions, we used a trimming and correction method. The results showed that the association between PNI and OS remained statistically significant after trimming, and the estimated HR was consistent with the direction and strength of the original pooled results. This result strengthens the reliability of our primary conclusions. Furthermore, our leave-one-out sensitivity analyses for the three outcome measures—OS, RFS, and DFS—all showed that removing any individual study did not lead to a directional reversal of the pooled HR, further confirming the robustness of our meta-analysis results.

The prognostic value of PNI may be related to several biological mechanisms. First, its core component, serum albumin, is a key indicator reflecting the body’s nutritional status and liver synthetic function (40–42). Malnutrition is directly associated with increased postoperative complication rates, decreased tissue repair capacity, and reduced tolerance to surgical stress (43, 44). In HCC patients, primary liver disease is often accompanied by varying degrees of liver dysfunction and synthetic impairment, further exacerbating the risk of malnutrition (45, 46). Second, lymphocyte count represents the state of the adaptive immune system (47). In the tumor microenvironment, lymphocytes are the main executors of the anti-tumor immune response (48). A higher lymphocyte count may indicate more active immune surveillance and a stronger ability to clear postoperative residual microlesions or circulating tumor cells (49). Therefore, a low preoperative PNI suggests that the patient is in a state of “nutritional-immune” dual depletion. This state may weaken the patient’s benefit from radical surgery, making them more susceptible to early recurrence and death.

Compared to other indicators, the unique advantages of the PNI lie in its simplicity, low cost, and comprehensive information (13). The serum albumin and lymphocyte counts required for PNI are routine clinical tests, easily obtainable and reproducible. Compared to single inflammatory or nutritional markers, PNI captures information from both nutritional and immune levels, potentially providing a more comprehensive profile of host status (21, 39). Our results support the prognostic value of PNI in this patient population, which is non-inferior to and may even be superior to some single indicators. However, future research could further explore combinations of PNI with other promising indicators to construct more robust prognostic prediction models, enabling more precise individualized prognostic stratification.

Although this study has confirmed a significant association between PNI and the survival outcomes after HCC liver resection, caution is necessary when evaluating its clinical practicality. Firstly, as PNI is a composite indicator consisting of albumin and lymphocyte counts, its prognostic information may partially overlap with existing liver function assessment tools and tumor staging systems. Currently, there is a lack of direct evidence indicating that PNI can provide significant incremental predictive value for these established prognostic models. Secondly, the clinical interpretation of PNI faces the issue of inconsistent cutoff values, which limits its application as a standardized risk stratification tool. Therefore, at this stage, PNI is more suitable to be regarded as an auxiliary indicator for preoperative risk screening, used to identify high-risk patients who require enhanced perioperative nutritional support and postoperative monitoring, rather than replacing the existing comprehensive prognostic assessment system. Future research should focus on verifying in prospective cohorts whether PNI can provide independent incremental information beyond traditional prognostic factors and explore its practical clinical utility when integrated into multivariate prediction models.

Patients with HCC often experience varying degrees of malnutrition and metabolic disorders, with etiologies involving multiple mechanisms, including anabolism due to liver dysfunction, tumor-associated catabolism hyperactivity, and gastrointestinal symptoms caused by portal hypertension (50). In recent years, diagnostic criteria advocated by the Global Nutrition Leadership Initiative have been applied to the nutritional assessment of HCC patients, demonstrating predictive value for post-hepatectomy prognosis (51). Furthermore, the development trend of multidimensional prognostic tools suggests that combining inflammation-nutrition composite indicators such as PNI with liver function assessment tools (e.g., ALBI classification) or imaging parameters (e.g., sarcopenia assessment) may lead to more accurate prognostic prediction models (52). Future research should explore the synergistic value of PNI with other nutritional and functional indicators to promote the individualization of perioperative nutritional management for HCC patients.

Of course, this study also has some limitations. First, all included studies were retrospective observational studies. Although most studies were of high quality, inherent selection bias and confounding bias could not be completely avoided. For example, the association between PNI and prognosis may be affected by other unmeasured or insufficiently adjusted confounding factors. Second, the lack of uniformity in the cutoff value of PNI limits its direct application to clinical decision-making, and more prospective studies are needed to determine the optimal cutoff value. Third, our analysis was mainly based on aggregated data from published literature, and meta-analysis of individual patient data was not possible, making it difficult to conduct more in-depth subgroup analyses. Fourth, due to incomplete reporting of key covariates in the original studies, this study was unable to conduct a meta-regression analysis, an inherent limitation of the current evidence base that also highlights the need for standardized reporting in future primary research. Future research needs to conduct more large-scale, multicenter prospective cohort studies to validate the prognostic value of PNI, while determining a standard cutoff value and exploring its relationship with treatment response as a gold standard. In addition, whether dynamic changes in PNI have stronger prognostic predictive value than a single preoperative value is also a direction worthy of further investigation.

## 5 Conclusion

This systematic review and meta-analysis suggests that a higher preoperative PNI is a potential protective factor for long-term survival in HCC patients undergoing hepatectomy. Despite some inter-study heterogeneity, sensitivity analysis confirmed the stability of the results, and adjustments for publication bias did not alter the core findings. Further prospective studies are warranted to validate the optimal cutoff value and to assess the incremental predictive value of PNI over established prognostic models.

## Data Availability

The datasets presented in this study can be found in online repositories. The names of the repository/repositories and accession number(s) can be found in the article/**Supplementary Material**.
