# Supplementary material for: Prognostic value of prognostic nutritional index in patients with hepatocellular carcinoma undergoing hepatectomy: a systematic review and meta-analysis
Source: Front Oncol. 2026 Jul 29;16:1887443. doi: 10.3389/fonc.2026.1887443 (PMC13461806; doi:10.3389/fonc.2026.1887443)
Supplement: Supplementary file 1 [file DataSheet1.docx]

**TableS1 Literature Search Strategy**

Pubmed-225

(("Carcinoma, Hepatocellular"[Mesh]) OR ((((Hepatocellular Carcinoma) OR (Hepatoma)) OR (Adult Liver Cancer)) OR (Liver Cell Carcinoma))) AND ((prognostic nutritional index[Title/Abstract]) OR (PNI))

Embase-335 (Via OVID)

((Carcinoma, Hepatocellular or (Hepatocellular Carcinoma or Hepatoma or Adult Liver Cancer or Liver Cell Carcinoma)) and (prognostic nutritional index or PNI)).af.

Cochrane-3 (Via OVID)

((Carcinoma, Hepatocellular or (Hepatocellular Carcinoma or Hepatoma or Adult Liver Cancer or Liver Cell Carcinoma)) and (prognostic nutritional index or PNI)).af.

WOS-397

((Carcinoma, Hepatocellular) OR ((((Hepatocellular Carcinoma) OR (Hepatoma)) OR (Adult Liver Cancer)) OR (Liver Cell Carcinoma))) AND ((prognostic nutritional index) OR (PNI)) (All Fields)
